# Sensitivity to Emotion Intensity and Recognition of Emotion Expression in Neurotypical Children

**DOI:** 10.3390/children8121108

**Published:** 2021-12-01

**Authors:** Koviljka Barisnikov, Marine Thomasson, Jennyfer Stutzmann, Fleur Lejeune

**Affiliations:** Department of Psychology, FPSE, University of Geneva, 1205 Geneva, Switzerland; Koviljka.Barisnikov@unige.ch (K.B.); Marine.Thomasson@unige.ch (M.T.); Jennyfer.Stutzmann@unige.ch (J.S.)

**Keywords:** emotion expression recognition, emotion intensity, face emotion processing, neurotypical children

## Abstract

This study assessed two components of face emotion processing: emotion recognition and sensitivity to intensity of emotion expressions and their relation in children age 4 to 12 (N = 216). Results indicated a slower development in the accurate decoding of low intensity expressions compared to high intensity. Between age 4 and 12, children discriminated high intensity expressions better than low ones. The intensity of expression had a stronger impact on overall face expression recognition. High intensity happiness was better recognized than low intensity up to age 11, while children 4 to 12 had difficulties discriminating between high and low intensity sadness. Our results suggest that sensitivity to low intensity expressions acts as a complementary mediator between age and emotion expression recognition, while this was not the case for the recognition of high intensity expressions. These results could help in the development of specific interventions for populations presenting socio-cognitive and emotion difficulties.

## 1. Introduction

The ability to recognize emotion expressions is fundamental for processing social information that supports the development of socio-cognitive abilities and daily social interaction [1,2,3]. Many studies have reported age-related changes in emotion recognition abilities with an unequal developmental pattern across emotions. The recognition of the happy face expression develops earlier, while the recognition of sadness and anger largely improve between age 5 and 7; surprise is identified between age 6 and 10, and fear after age 10 [4,5,6]. These results also varied according to the task demand. Children as young as 6 show high performances on labeling tasks, while on matching tasks a similar result was not observed before age 10 [7,8,9,10,11]. The recognition of fear develops even later, with low success in younger children on labeling and matching tasks [12,13]. The unequal across emotions recognition pattern may result from the development of ability to detect more complex and discrete facial expressions with age [1,14]. However, most developmental studies used photographs of intense facial expressions and recognition of discrete expressions were less studied in the children population. In everyday life, we are more frequently exposed to less intense facial expressions that may affect perception and interpretation of emotion feeling. Seeing someone with a lower intensity sad expression should result in a different reaction than when seeing an intense expression of sadness, for example. Sensitivity to different intensities of emotion expressions could help to infer others’ feelings more precisely and in turn to develop prosocially oriented behavior [2,15,16]. The present manuscript will focus on age-related changes regarding recognition intensity of face emotion expression.

To evaluate the recognition of more discrete facial emotion expressions, studies in children and adults used a morphing technique to simulate facial muscular movement during a particular expression. This is achieved by progressively moving the position of the features from the neutral face expression over several sequences, usually varying by 5% to 10%, toward their position in an intense emotional face expression (100%) or above [17]. Several studies have been conducted in neurotypical (NT) adults reporting that older adults present more difficulties compared to younger ones in naming or matching low intensity face emotion expressions, 50%, but not for high intensity, 100% [18,19,20,21]. A few developmental studies used morphed facial expressions and showed that young children had more difficulties in accurately decoding subtle expressions. One study [22] used four levels of emotion intensity (25%, 50%, 75% and 100%) in children age 4 to 15 years. Results showed no relation between age and level of intensity, but differences in performances regarding the lowest and highest intensities of emotion expressions (sadness, anger, happiness, fear and disgust) were observed. Authors concluded that intensifications were too narrow to apprehend the variations in the mid-range intensity. Some authors used even the finest intensity measures (20 levels) with 5% of increment [23,24]. The results indicated that young children recognized low intensities of happiness but were less accurate at identifying sadness and fearful expressions until age 10, even for higher intensity (100%). The recognition of surprise, disgust, and fear continued to improve between age 5 and 10 years, but an increase in sensitivity to sadness and anger was observed even after age 10, and into adulthood. Thomas, De Bellis, Graham and LaBar [25] used six intermediate intensities (at 11 degrees) and asked participants to decide if faces expressed emotion or not (angry versus neutral; fearful versus neutral). Results showed a significant difference only between adults (age 25 to 57) and children (age 7–13) regarding fear expression, and between adults, children and adolescents (aged 14 to 18 years) regarding anger expression. Although the results provided some new information, the difference between age groups was too wide and intensity increments too narrow to identify variation across childhood and adolescence. Finally, Rodger, Lao and Caldara [26] used a psychophysiological approach to study the developmental trajectory regarding the emotion expression recognition, from age 6 up to adulthood. They first assessed the number of signals necessary to recognize expressions at high intensity; secondly, they quantified the level of expression intensity necessary for each participant to recognize an emotional expression. Both measures revealed that happiness was the easiest to recognize for all age groups, while fear showed to be more difficult. The recognition of other emotions (sadness, anger, disgust, and surprise) develops progressively, showing a decrease with age in the quantity of signal and intensity required to recognize these expressions. The analysis also indicated that intensity and signal processing are similar only during adulthood. The authors concluded that the developmental trajectory during childhood for recognition of full-intensity emotional expressions might be different from the recognition of varied-intensity expressions.

As seen above, developmental studies pointed out that the sensitivity in perceiving subtle changes in facial emotion expressions has a slower developmental course than for recognition of intense expressions. The distinction between the two components of emotion perception, emotion recognition and sensitivity to emotion intensity [20], seems to be supported by different neuronal mechanisms [27,28,29]. However, as seen above, studies reported stronger age-related differences for recognition of full-intensity expressions for some emotion (e.g., sadness, surprise, fear) and for sensitivity to low intensity expressions. Such observation should encourage assessing the relation between these two components, in order to examine their possible interdependence during development. This is of great importance as from a behavior point of view, the difficulties in perception of discrete emotion expressions could interfere in young children’s interpretation of emotion cues. Decoding errors could mislead understanding of social situations and in turn initiate non-adapted responses to a particular situation. These difficulties may be of particular relevance for children with neurodevelopmental disorders associated with psychopathological and socio-emotion difficulties [1,30,31,32,33].

Considering the importance of the subject, study on developmental trends in emotion sensitivity is warranted, and in particular on relations between face emotion recognition and sensitivity to intensity of expression in the same child population. However, some methodological issues must be considered when studying child populations. Stimuli with several intensity levels seem particularly difficult and little informative for studying young children and those with developmental disorders [34,35] as well as for older adults [36]. They could challenge attentional abilities and perception of discrete visual changes [23], and seriously impact children’s performances. As seen above, two levels of intensity (low and high) and the tasks with a reduced number of possible response options seems more sensitive to age-related changes [37]. It also appears that the emotion recognition tasks not relying on language and memory abilities are more suitable for young children and those with developmental disabilities [38,39,40]. Despite these observations, we still need adapted tasks for assessing the sensitivity of emotion expression intensity in these populations. In one comparative study in adults with Down syndrome (DS), Hippolyte, Barisnikov, Van der Linden and Detraux [41] assessed two components of face emotion processing: (1) the recognition of basic high intensity face emotion expressions, adapted from the Bruce et al.’s (2000) battery; (2) the discrimination between low and high intensity expressions (happy versus sad versus neutral), with The facial discrimination task FDT; [42]. Results showed that the adults with DS differed from the control NT children only on the recognition of high intensity sad expressions. Additionally, the NT children obtained significantly better scores for the identification of happy and sad expressions of high intensity compared to low intensity of these emotions. In their second experiment, the authors showed specific relations between scores on the recognition of basic emotion expressions and the ability to attribute emotion feelings according to the context (emotion attribution task) in DS adults as well as in NT children.

The FDT task proved to be well adapted for assessing sensitivity to intensity of emotion expressions in young NT children and adults with DS. However, information about the developmental course of these abilities was rather limited, as the study included only NT children age 4 to 7 years in comparison with adults with DS. Consequently, it is of importance to assess a wider age-range of a NT population. It would allow detecting critical periods for the development of sensitivity to lower intensities of emotion expressions and its relation to the recognition of high intensity basic emotion expressions throughout childhood. Based on a literature review, one could suggest that higher performances in recognition of some basic face emotion expressions could be related to an improvement in the ability to detect more subtle changes in face expressions. In other word, more precise processing of facial expressions is needed to distinguish between emotions that share some physical features (e.g., the mouth for happiness vs. sadness; the eyes for fear vs. surprise), for example. The ability to detect low intensity expression could be seen as one explanatory factor for the improvement in recognition of different face emotion expressions with age.

The present research has two main aims: (1) to assess the recognition of basic face emotion expressions and the ability to discriminate between two levels of emotion expression intensity, in children age 4 to 12 and adults; (2) to assess relations between these two components of face emotion processing abilities. We expect that better recognition of low intensity expressions will be associated with higher performances on emotion recognition tasks and that emotion intensity recognition would mediate the relationship between the age and the emotion recognition performances.

## 2. Methods

### 2.1. Participants

Two hundred and sixteen NT children age 4 to 12, without developmental and learning difficulties, participated in the study. The required number of participants in each group has been determined by a power analysis involving the comparison of two means: $N=\frac{2\;\times \;\sigma^{2}\;\left(z_{\frac{\alpha }{2}}+z_{\beta }\right)\;}{{\left({\bar{X}}_{1}-{\bar{X}}_{2}\right)}^{2}}$. This analysis was based on a previous study that had investigated the capacity to process facial expressions in typical developed children and adults with Down syndrome [41]. To achieve the desired statistical power (1 − *β*) of 90% and risk of Type I error (*α*) of 0.05, results indicated that for a one-sided hypothesis, 12 participants would be needed in each group.They were separated into nine age groups of 24 each, and one group composed of 14 adults. Children and adults with lower raw PPVT-R score, according to the norms from Dunn, Thériault-Whalen and Dunn [43], were excluded from the study. The population description is shown in Table 1. Chi-square analysis indicated no significant difference regarding gender among age groups, χ^2^(9) = 6.50, *p* = 0.69.

Children were recruited via local primary schools in the city of Geneva after receiving the parents’ consent form about the study, accordingly to Helsinki declaration [44]. The Ethical Committee of the Department of Psychology at the University of Geneva and the Cantonal Authorities for Primary Education delivered the authorization (accepted in September 2009; n°E-0409-2009/12). All participants were volunteers and could leave the study at any time.

### 2.2. Procedure

All participants were assessed with two tasks adapted from the face processing tests battery [8] by Hippolyte, et al. [41], as well as with the facial discrimination task [42] and the Peabody picture vocabulary test-revised, adapted for the French-speaking population [43].

They were assessed individually by an experienced psychologist in a quiet room. The child participants were assessed at their school, and adults in our laboratory at the University of Geneva. The different tasks were administrated in a pseudo-random order in two to four sessions lasting about 20 min each.

#### 2.2.1. Recognition of Face Expressions

The recognition of high intensity face emotion expressions (happy, sadness, anger, surprise and neutral) was assessed with the expression identification and the expression matching tasks adapted from two tests (emotion-id and emotion-match, respectively) of the Bruce et al.’s (2000) battery. A distractor (two instead of one) and the neutral expression were added [41]. An additional item per facial expression for the identification task was also added to increase the task demand [1]. All stimuli consisted of monochrome photographs of children’s and adults’ faces (5.5 cm × 4 cm), presented with a uniform grey background.

*Emotion identification task.* This modified task consisting of 20 items (instead of twelve) and one trial item, with four items per expression (instead of three). There was also a higher number of distractors (two instead of one) to increase the task demand and avoid a ceiling effect. Each participant was shown the three faces, one next to the other, and they had to indicate the face that displayed a particular expression named by the experimenter (happiness, sadness, anger, surprise or neutral).

*Emotion matching task.* This task was composed of two parts: one with children’s faces and the other with adults’ faces (15 items each and one trial item). A target stimulus was presented at the top of the page and the participant had to identify the face at the bottom (out of three faces) that showed the same expressions (happiness, sadness, anger, surprise and neutral; 3 items per expression). The percentage of correct answers for predicted emotion, established by [8] for each task and each emotion, was calculated. Moreover, a global emotion expression score (sum of the two subtests) was calculated.

#### 2.2.2. Expression Intensity Discrimination Task

The *facial discrimination task.* FDT [42] evaluated the ability to discriminate between high and low intensity emotion expressions. It consisted of 41 black and white faces’ photographs (13 cm × 18.5 cm), with happy, sad or neutral expressions. For each item, the participants had to indicate whether a given item depicted a happy face, a sad face, or a face that was neither happy nor sad (neutral). If the response was happy or sad, they were asked to decide between two intensity levels for that emotion. Level 1 (low intensity) was for a face that was ‘a little’ happy or sad (50% intensity) and level 2 (high intensity) for a face that was ‘very’ happy or sad (100% intensity). The participants had to point at either a small vertical column that represented a low intensity emotion or a large vertical column that represented a high intensity emotion. The task began with a training session of six items and the test was composed of 35 items, with 12 happy faces (9 low intensity, 3 high intensity), 11 sad faces (7 low intensity, 4 high intensity) and 12 neutral faces. The faces were presented in a counterbalanced order. A global expression discrimination score was calculated, and included the percentage of correct answers for all 35 items. Emotion intensity scores were calculated separately for each emotion expression (sad and happy) for each intensity (low and high).

A global emotion intensity score for high intensity (happy and sad) and a global emotion intensity score for low intensity (happy and sad) were also calculated.

#### 2.2.3. Peabody Picture Vocabulary Test-Revised (PPVT-R)

A French adaptation of the PPVT-R vocabulary scale [43] was selected to evaluate the receptive vocabulary. For each item, composed of four pictures, the participants had to indicate the picture corresponding to a word named by experimenter. The test administration was stopped after six erroneous responses over eight consecutive trials. The raw vocabulary score was calculated.

### 2.3. Statistical Analysis

Statistical analyses were conducted using Statistica and SPSS. For each total score of each task, repeated-measure analyses of variance (ANOVA) were performed with the percentage of correct answers as a dependent variable. As the homogeneity of variance assumption needed for an ANOVA was not always respected (Table S1), we performed Huynd-Feldt correction estimates epsilon in order to correct the degrees of freedom of the F-distribution.

Firstly, we investigated the developmental changes in face emotion processing, in order to identify and compare critical periods of development assessed by three tasks. A repeated-measures ANOVA was done with the percentage of correct answers as the dependent variable, with 10 age groups (nine groups age 4 to 12 and one adult group) and Gender (girl, boy) as two between-subject factors, and Task (emotion identification, emotion matching and facial discrimination task) as a within-subject factor. Post hoc comparisons were performed by using Bonferroni analyses. Being more rigorous than the Tukey’s test (which tolerates type I errors) and more generous than the very conservative Scheffé’s method, we chose the Bonferroni correction method [45]. Moreover, the Bonferroni post hoc test produces the narrowest confidence intervals, which means it has the greatest ability to detect true difference between our groups of interest.

As identification and matching tasks assessed the emotion recognition of five same-face expressions, a repeated-measure ANOVA was conducted with the age groups as a between-subject factor (age 4 to 12 and adults), task (Identification vs. Matching) and emotion (happiness, sadness, anger, surprise versus neutral) as within-subject factors.

For the facial discrimination task, a first repeated-measure ANOVA was conducted with the global expression discrimination score as a dependent variable, with age groups (Age 4 to 12 and Adults) and gender (girl, boy) as between-subject factors and emotion (happiness, sadness vs. neutral) as a within-subject factor. The second one was performed with the global emotion intensity score as a dependent variable, age groups (Age 4 to 12 and Adults) and gender (girl, boy) as between-subject factors and two within-subject factors: emotion (happiness and sadness) and intensity (Level 1 and 2). Post hoc comparisons were made using the Bonferroni test.

Finally, we conducted mediation analysis in order to assess the extent to which performances to attribute low or high intensity acted as a link between the age of the participant and the emotional performances (i.e., global emotion expression score). To do so, we ran a bootstrapping analysis using the SPSS process macro developed by Hayes [46].

## 3. Results

### 3.1. Task Comparison

A significant effect of age, *F*(9,229) = 27.62, *p* < 0.001, η^2^_p_ = 0.52 was found. 5-year-olds had lower performances on the three tasks (M = 77.6%, SD = 1.3) than 6-year-olds (M = 86.6%, SD = 1.2). No significant difference between two consecutive age groups was revealed after age 6, indicating a linear improvement of performances from age 6 to adulthood. Results also revealed a significant effect of task, *F*(2,460) = 43.91, *p* < 0.001, η^2^_p_ = 0.16. Worse performances were observed for the emotion matching task (M = 85.20%, SD = 0.6) in comparison with the performances obtained for the facial discrimination task (M = 90.50%, SD = 0.6) and for the emotion identification task (M = 90.82%, SD = 0.5) (*p* < 0.001). However, no significant difference between the performances in the emotion identification task and the facial discrimination task was observed. There was also a significant task x age interaction, *F*(18,460)= 2.65, *p* < 0.001, η^2^_p_ = 0.09. 4-, 6-, and 7-year-olds had worse performances on the emotion matching task than the two other tasks (emotion identification and facial discrimination task) (*p* < 0.01), but no difference was observed between the emotion identification and the facial discrimination task. The 5-year-old children had better performances on the emotion identification task than on the emotion matching task (*p* < 0.001) and the facial discrimination task (*p* = 0.04), but no difference was observed between the emotion matching and facial discrimination task. Any significant difference was observed among the three tasks from age 8 to adulthood.

A significant effect of gender was found, *F*(1,229) = 5.82, *p* = 0.02, η^2^_p_ = 0.03: girls had better scores than boys (*p* < 0.001). There was no other significant effect.

### 3.2. Emotion Recognition

The results are presented in Table 2, as well as in the Supplementary Materials (Figures S1–S3). The results revealed a significant effect of the age groups, F(9,230) = 20.88, *p* < 0.001, η^2^_p_ = 0.45. Post hoc analyses did not reveal any significant difference between two consecutive age groups, indicating a linear improvement of performances from age 4 to adulthood. A significant effect of the task was found, F(1,230) = 76.04, *p* = 0.01, as well as a significant age groups x task interaction, F(9,230) = 3.35, *p =* 0.04, η^2^_p_ = 0.03. Post hoc analyses revealed that 4-, 5-, 6-, 7- and 9-year-olds had better performances on the ID task than on the matching task. A significant effect of emotion was observed, F(4,920) = 26.09, *p* < 0.001, η^2^_p_ = 0.20, as well as a significant age groups x emotion interaction, F(36,920) = 3.03, *p < 0*.001, η^2^_p_ = 0.11. Post hoc analyses showed that happiness is better recognized than all other emotions (all *p* < 0.01) at age 4, 5 and 6. Moreover, at age 5, surprise is better recognized than neutral (*p* < 0.001). Finally, a significant effect task x emotion interaction was observed, F(4,920) = 39.10, *p* < 0.001, η^2^_p_ = 0.10, as well as a significant age group x task x emotion interaction, *F*(36,920) = 2.56, *p* < 0.001, η^2^_p_ = 0.10. Post hoc analyses demonstrated that for anger, 4-, 5- and 6-year-old children had better scores on the ID task than the matching task (all *p* < 0.001); this difference disappeared as of age 7. For surprise, 4- and 6-year-old children had more difficulties on the matching task than on the ID task (all *p* < 0.05). For neutral, 5-year-olds had better scores for the ID task than for the matching task (*p* < 0.05).

### 3.3. Expression Intensity Discrimination

The resuslts of the facial discrimination task are presented in Table 3, as well as in the Supplementary Materials (Figures S4–S6), and they showed:

- For the global expression discrimination score, results revealed a significant effect of the age groups, F(9,229) = 16.61, *p* < 0.001, η^2^_p_ = 0.40. Children were better at age 6 than 5 (*p* < 0.001). No significant effect of the emotion was found, F(2,373) = 0.81, *p* = 0.34, η^2^_p_ = 0.004, but a significant age groups x emotion interaction was observed, F(15,373) = 0.81, *p* < 0.001, η^2^_p_ = 0.11. The post hoc analysis showed that 4-year-old children recognized the neutral expression better than sadness (*p* = 0.01) and that 5-year-old children recognized happiness better than the neutral expression (*p* < 0.001). The gender factor was not significant, F(1,384) = 0.81, *p* = 0.0.09, η^2^_p_ = 0.01. There was no other significant effect.
- For the global emotion intensity score, results showed a significant effect of age groups, F(9,229) = 13.58, *p* < 0.001, η^2^_p_ = 0.35. Post hoc analyses revealed that 6-year-old children were better at discriminating emotion intensity than 5-year-old children (*p* < 0.001). The results showed a significant effect of intensity, F(1,230) = 224.49, *p* = 0.002, η^2^_p_ = 0.04, as well as a significant effect of age group x intensity interaction, F(9,230) = 385, *p* < 0.001, η^2^_p_ = 0.04. Post hoc analyses revealed that 4-, 5-, 6-, 7-, 8-, 9-, 10- and 12-year-old children better identified high intensity emotions than those with low intensity (all *p* < 0.05). A significant effect of emotion was found, F(1,230) = 9.68, *p* = 0.002, η^2^_p_ = 0.49 and post hoc analyses demonstrated that sadness was better recognized than happiness (*p* = 0.002). Finally, a significant effect of age group x intensity x emotion interaction was also observed, F(9,230) = 2.14, *p* = 0.02, η^2^_p_ = 0.08. For happiness, children better recognized high intensity emotions than those with a low intensity until age 11 (all *p* < 0.01). For sadness, the children at all ages had difficulties discriminating between high and low intensity emotions (all *p* < 0.01), contrary to the adults. The gender factor was not significant, F(1,229) = 0.48, *p* = 0.49 but a significant gender x intensity interaction was observed (F(1,229) = 4.52, *p* = 0.03), where both boys and girls had better performances identifying high intensity emotions over low intensity ones. However, the calculation of effect sizes indicates that this difference was more pronounced among girls (Cohen’s d = −1.56) than boys (Cohen’s d = −1.21). There was no other significant effect.

### 3.4. Mediation Analyses

**Figure 1:**
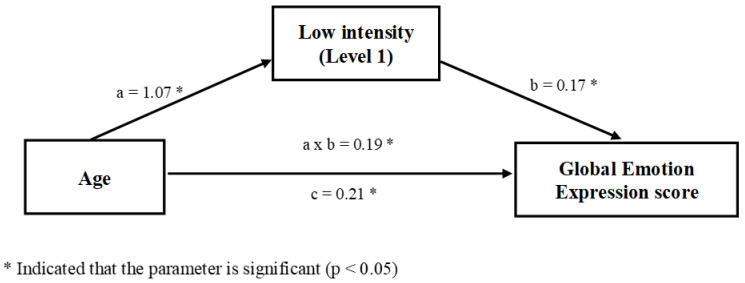
The ability to attribute low intensity emotional expressions mediates, in a complementary manner, the relationship between age and the global emotion expression score.

We first performed a mediation analysis in order to assess the extent to which performances to attribute low intensity (i.e., performances for expressions presenting with level 1 of intensity in the facial discrimination task) acted as a link between the age of the participants and the global emotion expression score (Figure 1). Most importantly, the relationship between the age and the score on global emotion recognition was mediated by the ability to attribute low intensity as attested by the significant indirect effect (a × b = 0.19; 95% CI [0.12, 0.29] not containing zero, which confirmed the existence of a mediation).

Moreover, as the Figure 1 illustrates, age had a strong influence on the ability to attribute low intensity (unstandardized regression coefficient a = 1.07, *p* < 0.001, 95% CI [0.77, 1.38], adjusted R^2^ = 0.09, F (1,228) = 24.84), suggesting that a higher age was related to better performance in attributing low intensity. Furthermore, better performances in attributing low intensity were related to a better global emotion recognition score (unstandardized regression coefficient b = 0.17, *p* < 0.001, CI [0.12, 0.22], adjusted R^2^ = 0.17, F(1,228) = 47.71). Overall, these findings suggest that a higher age lead to better performances in attributing low intensity emotions, which induced better performances in the emotional tasks. However, a direct effect of age (unstandardized regression coefficient c = 0.21, *p* = 0.001, 95% CI [0.08, 0.35], adjusted R^2^ = 0.20, F(1,228) = 60.08) was found when attributing low intensity emotion was excluded from the model, which suggests that there was an impact of the age per se on the global emotion expression score. Thus, these results support the assumption that the ability to attribute low intensity acts as a complementary mediator between age and the global emotion expression score.

**Figure 2:**
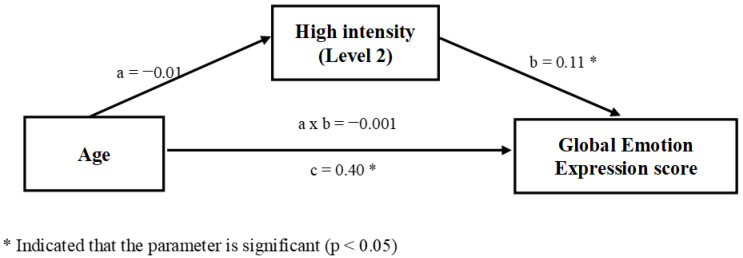
The ability to attribute high intensity emotional expressions was not a mediator of the relationship between age and the global emotion expression score.

Second, we assess the extent to which the ability to attribute high intensity (i.e., performances for expressions presenting with Level 2 of intensity on the FDT) acted as a link between age and the global emotion recognition score (Figure 2). Here, the relationship between those two variables was not mediated by the ability to attribute high intensity, as revealed by the non-significant indirect effect of age on the global emotion recognition score via performances to attribute high intensity (a × b = −0.001, 95% CI [−0.03, 0.03] containing zero).

Finally, we performed a mediation analysis with age and scores obtained on each emotion recognition task (the emotion identification task or the emotion matching task) with high/low intensity as a mediator. These results support the assumption that the ability to attribute low intensity acts as a complementary mediator between age and both emotion recognition tasks, but those relations were not mediated by the ability to attribute high intensity expressions. Results are available in the Supplementary data (Figures S7–S10).

## 4. Discussion

The present study aimed to assess the development of the two components of face expression processing: recognition of emotion expressions and sensitivity to the intensity of emotion expressions. We also examined the relation between these two components in large NT child populations age 4 to 12 and one adult group.

Overall, the results showed a significant improvement in face expression recognition with age for the three tasks. More specifically, the 5-year-olds had lower performances on the three tasks than the 6-year-olds, then a linear improvement from age 6 to adulthood was observed. A different developmental pattern for the emotion matching task was observed, showing that the children aged 4, 6, and 7 had significantly lower performances than on emotion identification and FDT. Finally, the 5-year-olds performed better on identification than matching and FDT, but no difference between emotion matching and FDT was observed. The differences among the three tasks disappeared from age 8 to adulthood. The absence of difference between identification and FDT could be explained by the task condition. In both tasks the emotion expression was named by the experimenter and the participants had to choose the corresponding expression among three faces (identification task) or among three labeled propositions made by the experimenter (FDT). According to the literature, success on these two tasks depends more on semantic representation, which develops early, while matching facial expressions depends more on visual-perception processing, which takes a longer maturation course [4,7,10].

One should keep in mind that we used a modified identification and matching task from the Bruce et al.’s [8] battery and introduced a higher number of distractors and items per emotion. This was similar to the Barisnikov, Theurel, et al.’s [1] study but with an added neutral expression. Our results showed a linear improvement from age 4 to adulthood, with higher performances on the identification task than those of the matching task at age 4, 5, 6, 7 and 9. In contrast, their results showed stage like pattern with a significant increase on the identification task from age 4/5 to 6/7 and on the matching task two periods of significant improvement at age 6/7 and 8/9 years. These differences could be related to methodological issues, as in the present study we examined performances year by year in children age 4 to 12, while Barisnikov, et al. [1] divided children age 4 to 11 into four age groups. The later one could have produced a developmental pattern that was more stage marked than what was shown by our study where more a linear improvement with age was observed. Investigating the development of an ability year by year in a large children population could be particularly useful for a comparative study with some clinical populations.

In addition, our results indicated that girls had better performances than boys on three tasks; this could be mainly related to their higher sensibility to the intensity of expression. Some prior studies have reported that girls have an advantage in recognizing emotion from facial cues [24,47,48] while others did not find a significant gender effect [23,49,50]. Studies in adults reported that women only recognized subtle emotional expressions better than men [51], but the intensity effect on gender seemed to depend on several factors, such as emotion valence, gender of displayed faces, level of expression intensity or participants’ emotional difficulties [52,53]. The gender effect on sensitivity to emotion intensity during development, need to be further studied to better understand its impact on socio-emotional behavior.

### 4.1. Emotion Expression Recognition

Regardless of the changes made in the identification and matching tasks, our results are in line with the literature, showing that the happiness is recognized earlier, followed by sadness and anger. In contrast, surprise is an emotion the recognition of which develops later, between age 6 and 10 and is therefore more difficult to identify and match [1,5,6,7,12,13]. The recognition of disgust and fear is reported to develop even later, with low success in younger children on labeling and matching tasks [9,12], but these emotions were not assessed in our study.

Our results also showed that at age 5, children had a better score for the neutral expression on the identification task than on the matching task; at age 5, surprise was better recognized than the neutral expression. Additionally, the results on the FDT task indicated that 4-year-old children recognized the neutral expression better than sadness, while 5-year-olds recognized happiness better than the neutral expression. These differences were only observed between age 4 and 5. Gao and Maurer [23] reported that 5-year-old children were as accurate as adults in distinguishing between the face expressing emotion and the neutral one. Introducing a higher number of items and distractors per emotion, however, may possibly have helped to avoid ceiling-level performances for the happy expression, which was not the case for some studies [8,24]. This is important as this effect proved to have a significant impact on the developmental trend of recognition of basic emotion expressions [32,54].

### 4.2. Emotion Intensity Recognition

Overall, the results revealed that 6-year-old children showed more sensitivity to discriminate between high and low intensity expressions than 5-year-olds. Furthermore, 4- to 10-year-olds and 12-year-olds identified emotions presented with a high intensity better than those with a low intensity, while no differences in the adult group were observed. Similarly, Gao and Maurer [23] reported that 5- to 7-year-olds, were significantly less sensitive than adults to lower emotion intensity expressions, reaching adult performances at age 10.

Consistent with the literature, our results also demonstrated that the impact of intensity level varied according to the type of emotion. Our children recognized high intensity happiness better than low intensity until age 11. Several studies have reported that children between age 5 and 6 recognized high intensity happy expressions similarly to adults [5], while sensitivity to lower intensity happy expressions develops between age 4 and 15 [35]. In contrast, Gao and Maurer [23] reported that, by age 5, children were as sensitive as adults, even for low intensity happiness expressions, reaching a ceiling-level performance from age 5. Regarding sadness, all our children (4 to 12 years old) had difficulties discriminating between high and low intensity expressions, contrary to the adult group. Gao and Maurer [23] reported that children were less accurate in identifying sad and fearful expressions until age 10, even for higher intensity, and only reached adults’ occurrence for fear at age 10. Rutter, et al. [32] reported that sensitivity to intensity for happiness, anger and fear expressions improved throughout adolescence and early adulthood, but sensitivity to anger expression developed in a steeper manner during early and mid-adolescence. According to the authors, their results strongly support specific life-span changes for each of the three emotions that could not be attributed to task-related confounds (e.g., ceiling effect for happiness recognition), unlike some previous studies [23]. A similar pattern of performances has been observed by a few studies that assessed all basic emotion expressions. Gao and Maurer [24] reported that sensitivity to low intensity sadness and anger expressions still improved even after age 10 and into adulthood, while sensitivity to intensity of surprise, disgust, and fear increased between age 5 and 10. Using a psychophysiological approach, Rodger, et al. [26] observed that recognition of lower intensity happiness expressions was the easiest to recognize, while fear was most difficult across all three adolescent groups (13–14, 15–16 and 17–18-year-olds) and the adult group. The authors concluded that this pattern proved to be stable from early childhood, but recognition of lower intensity expressions of sadness, anger, disgust and surprise develops progressively with age. Most importantly, the authors observed that, in contrast to adults, the childhood development for recognition of full intensity emotional expressions seems to follow a different trajectory from those with varied intensity. In line with the literature, our results indicated a slower development in the accurate decoding of low intensity facial expressions during childhood in comparison with high intensity expressions. This was true even for emotions that are recognized from an early age such as happiness.

Inferring subtle changes in face emotion expressions depends on highly efficient visual-perception processing that has a longer developmental trend [55] and is dissociated from processes involved in the recognition and labeling of emotion expressions [27,28,29]. Furthermore, different neuronal networks respond to variation in emotion intensity compared with changes in emotion category [27,56], which progressively mature with age, improving the ability to identify more complex face emotion cues [4,57]. Thus, a similar pattern of performances for the recognition of emotion expressions and sensitivity for their intensity are observed in adolescents and adults, as opposed to children [26,32].

Surprisingly, only few a research projects have assessed the ability to recognize intense and subtle expressions in the same child population while assuming that the latter could impact children’s efficiency in emotion processing abilities with age [32]. As far as we know, our study is the first that has directly investigated the impact of sensibility to the intensity of emotion expressions and emotion recognition ability during childhood.

### 4.3. Relation between Emotion Expressions and Emotion Intensity

Our mediation analyses showed that the link between age and the global emotion recognition score was mediated by the ability to identify a low intensity expression. Although age seems to have a more direct impact on the emotion recognition score, our results suggest that the ability to attribute low intensity acts as a complementary mediator between age and the global emotion expression recognition score during development (Figure 1). In contrast, this is not the case for the high intensity level (Figure 2). Further analysis also showed that the ability to attribute low intensity acts as a complementary mediator between age and each of the emotion recognition tasks (identification and matching), but these relations were not mediated by the ability to attribute high intensity expressions (see Supplementary Material). These results may suggest that the recognition of intense expression could be based on more global face processing that develop earlier, while ability to detect more subtle changes in face expressions demand more precise processing. Such an ability may help to distinguish between emotions that share some physical features (e.g., the eyes for fear vs. surprise) that are recognized later, but also to perceive a variation of expressions of the same emotion (e.g., mouth for happy) allowing recognition and understanding of their meaning (e.g., very happy/little happy).

Our results could be of particular interest to professionals and researchers working in the field of socio-emotion development. Indeed, improving the abilities to detect more subtle changes in face expressions could have a positive impact on socio-emotion development. It could support young children in their abilities to discriminate between different emotion expressions and in turn, elicit more adapted responses to some social situations [58,59]. It could also be interesting for older children to develop their abilities to decode low intensity expressions, as this would allow them to better identify and understand more subtle emotions. This could facilitate the development of their socio-emotion competences by supporting the development of positive peer interaction and friendship, as well as emotion regulation abilities [1,58,60].

Limitations on the generalizability of our findings should be acknowledged. First, it should be noted that we only assessed the relation between sensitivity to sad and happy expressions and recognition of basic emotion expressions. In addition, our children evaluated in this study were between 4 and 12 years of age but not later in adolescence. Our results suggest, however, that sensitivity to low intensity expressions remains complex at 12 years. Thus, further studies are needed to examine these relations during childhood and adolescence including a wide range of emotions. Furthermore, our study was cross-sectional and, as mentioned by Maxwell and Cole [61], cross-sectional data offer limited answers to questions about mechanisms underlying age-related changes. Consequently, our results cannot support the ability in emotion intensity recognition as a developmental mechanism (i.e., one caused by increasing age) or allow for conclusions about the causal relationships between age, performances in emotion intensity recognition, and performances in emotion expression recognition. Future investigations with longitudinal studies are required to further examine the causal association between the age and the performances in emotion expression recognition and the ability in emotion intensity recognition.

Nevertheless, a few studies that assessed the identification of lower intensity emotion expressions in children offer more nuanced comprehension of developmental trends of emotion recognition abilities during childhood. The sensitivity to a more subtle intensity of emotion expressions could help children to infer others’ feelings more precisely, which could be predictive of success in encoding emotion information [2,15,16].

This is of importance as decoding errors may mislead understanding of a situation and in turn initiate a non-adapted response to a particular event [40,59,62]. The ability to recognize more subtle face emotion expressions seems to extend beyond the ability to distinguish between different expressions. It involves a more complex representation and understanding of emotional cues that could impact the interpretation of the emotional state seen in the other’s face or in the specific social context [1,58], for example. In addition, several authors have suggested that emotion recognition abilities could have a modulation effect on the development of social-cognition (e.g., theory of mind, empathy) and on child behavior action and emotion regulation abilities [58,60]. Eggum, Eisenberg, Kao, Spinrad, Bolnick, et al. [63] suggested that emotion understanding might play a key role in the development of prosocial behavior. Barisnikov, et al. [1] revealed that good emotion recognition ability proved to be essential for effective emotion situation knowledge in NT children age 4 to 11. Nevertheless, difficulties in the recognition of more discrete expressions may be of particular relevance for children with neurodevelopmental disorders associated with psychopathological and socio-emotion difficulties [1,31,33,64]. Rutter, et al. [32] suggested that the development of a higher sensitivity to anger corresponds with a clinical risk period for externalizing pathology in adolescents.

However, we still need more studies that investigate the relation between emotion recognition and sensitivity to the intensity of emotion expressions during development for a better understanding of mechanisms underlying emotion processing. This could also bring a new insight concerning socio-cognitive and emotional difficulties in children’s clinical populations. Most importantly, our results highlighted the importance of studying jointly the processing of emotion expressions and the sensitivity to their intensity during development, which may share some common processes. Clarifying the origin of their relation could open a new perspective to explain the learning of emotion information.

## 5. Conclusions

Our results are in accordance with most studies confirming that recognition of high intensity face emotion expressions significantly improves with age and that an uneven developmental pattern across emotions was impacted by the task modality between age 4 and 6. Results also indicated a slower development of accurate decoding of low intensity expressions compared to high intensity. The children aged 4 to 10 and 12-year-olds identified high intensity emotion expressions significantly better than those of low intensity. Furthermore, the high intensity of happiness was better recognized than low intensity until age 11, while children age 4 to 12 had difficulties discriminating between high and low intensities of sadness.

Most importantly, our results suggest that sensitivity to low intensity expressions acts as a complementary mediator between age and the global emotion expression recognition score, which was not the case for recognition of high intensity expressions. These results could also help the development of targeted interventions for children with socio-cognitive and emotion difficulties.

## Figures and Tables

**Table 1:** Participants’ characteristics.

Age Groups	N	Age; M(SD)	Gender; % Girls	PPVT-R (Raw Score); M(SD)
Age 4	24	4.57 (0.17)	45.8	55.2 (15.2)
Age 5	24	5.10 (0.15)	37.5	64.7 (16.6)
Age 6	24	6.62 (0.31)	50.0	84.1 (18.3)
Age 7	24	7.71 (0.26)	45.8	101.2 (18.6)
Age 8	24	8.71 (0.31)	45.8	104.9 (14.1)
Age 9	24	9.56 (0.33)	50.0	115.0 (9.8)
Age 10	24	10.52 (0.32)	45.8	116.9 (14.1)
Age 11	24	11.45 (0.29)	50.0	124.4 (11.1)
Age 12	24	12.44 (0.26)	50.0	131.5 (7.1)
Adults	14	28.36 (6.65)	78.6	158.1 (8.7)

**Table 2:** Mean scores corrects (percentage), standard deviations in parentheses and global score for emotion expression tests according to the conditions emotion (happiness, sadness, anger, surprise and neutral) task (identification vs. matching) and age groups (4 to adults).

Age Groups/Task		Age 4 (N = 24)	Age 5 (N = 24)	Age 6 (N = 24)	Age 7 (N = 24)	Age 8 (N = 24)	Age 9 (N = 24)	Age 10 (N = 24)	Age 11 (N = 24)	Age 12 (N = 24)	Adults (N = 14)
Emotion recognition task		M% (SD)	M% (SD)	M% (SD)	M% (SD)	M% (SD)	M% (SD)	M% (SD)	M% (SD)	M% (SD)	M% (SD)
Happiness	ID	85.4 (2.8)	93.7 (2.8)	94.8 (2.8)	88.5 (2.8)	92.7 (2.8)	96.9 (2.8)	97.9 (2.8)	91.7 (2.8)	86.4 (2.8)	98.2 (3.7)
	Match	86.8 (2.1)	91.0 (2.1)	93.7 (2.1)	91.0 (2.1)	89.6 (2.1)	94.4 (2.1)	95.1 (2.1)	97.9 (2.1)	97.2 (2.1)	100 (2.7)
Sadness	ID	67.7 (3.9)	78.1 (3.9)	80.2 (3.9)	92.7 (4.0)	90.6 (3.9)	93.7 (3.9)	93.7 (3.9)	85.4 (3.9)	98.9 (3.9)	96.4 (5.2)
	Match	84.7 (2.2)	79.9 (2.2)	91.0 (2.2)	93.1 (2.2)	97.9 (2.2)	92.4 (2.2)	91.0 (2.2)	97.9 (2.2)	90.3 (2.2)	97.6 (2.9)
Anger	ID	94.8 (1.9)	92.7 (1.9)	96.9 (1.9)	94.8 (1.9)	97.9 (1.9)	97.9 (1.9)	92.7 (1.9)	98.9 (1.9)	98.9 (1.9)	94.6 (2.6)
	Match	61.8 (3.7)	70.1 (3.7)	68.7 (3.7)	78.5 (3.7)	84.0 (3.7)	86.1 (3.7)	89.6 (3.7)	87.5 (3.7)	89.6 (3.7)	96.4 (4.8)
Surprise	ID	79.2 (3.7)	82.3 (3.7)	95.8 (3.7)	95.8 (3.7)	89.6 (3.7)	100.0 (3.7)	94.8 (3.7)	91.7 (3.7)	93.7 (3.7)	98.2 (4.8)
	Match	56.2 (3.4)	65.3 (3.4)	68.7 (3.4)	77.8 (3.4)	82.6 (3.7)	84.7 (3.4)	81.9 (3.4)	88.9 (3.4)	85.4 (3.4)	94.0 (4.4)
Neutral	ID	67.8 (3.9)	70.8 (3.9)	80.2 (3.9)	88.5 (3.9)	86.4 (3.9)	87.5 (3.9)	92.7 (3.9)	96.9 (3.9)	91.7 (3.9)	94.6 (5.1)
	Match	62.5 (4.5)	61.1 (4.5)	77.8 (4.5)	81.9 (4.5)	84.7 (4.5)	77.8 (4.5)	94.4 (4.5)	95.8 (4.5)	88.9 (4.5)	92.8 (5.9)
Global Identification score		78.9 (12.7)	83.5 (10.6)	89.8 (7.7)	92.1 (4.1)	91.4 (6.8)	95.2 (5.6)	94.4 (6.6)	92.9 (9.3)	93.9 (5.7)	96.4 (4.1)
Global Matching score		70.4 (10.9)	73.5 (8.1)	80.0 (12.3)	84.4 (14.2)	87.8 (8.1)	87.1 (8.0)	90.4 (7.4)	93.6 (7.7)	90.3 (7.5)	96.2 (4.5)
Global Emotion Expression Score		74.7 (9.1)	78.5 (7.8)	85.0 (8.2)	88.3 (8.1)	89.6 (5.9)	91.1 (4.8)	92.4 (6.1)	93.3 (8.4)	92.1 (5.4)	96.3 (3.6)

**Table 3:** Mean scores corrects (percentage) and standard deviations in parentheses for the facial discrimination task according to the conditions emotion (happiness, sadness, and neutral), and to the intensity (low vs. high).

Age Groups/Task	Age 4 (N = 24)	Age 5 (N = 24)	Age 6 (N = 24)	Age 7 (N = 24)	Age 8 (N = 24)	Age 9 (N = 24)	Age 10 (N = 24)	Age 11 (N = 24)	Age 12 (N = 24)	Adults (N = 14)
Happiness	84.0 (12.5)	83.3 (19.6)	91.3 (5.2)	91.3 (13.8)	87.5 (8.1)	91.3 (4.6)	93.7 (4.4)	92.4 (4.2)	92.7 (6.6)	97.6 (3.9)
Sadness	71.2 (23.8)	75.0 (16.8)	91.3 (10.2)	93.6 (10.2)	86.0 (12.3)	93.2 (8.1)	96.6 (5.2)	94.7 (7.0)	97.3 (5.7)	97.4 (7.5)
Neutral	86.8 (21.3)	67.4 (37.0)	87.5 (26.5)	94.4 (10.0)	94.4 (9.1)	96.2 (7.8)	94.8 (10.4)	97.6 (5.2)	96.5 (4.9)	99.4 (2.2)
Global expression discrimination score	80.7 (13.8)	75.2 (16.4)	90.0 (8.6)	93.1 (7.3)	89.3 (7.8)	93.6 (4.2)	95.0 (3.6)	94.9 (3.3)	95.5 (4.6)	97.5 (3.4)
Emotion intensity										
Happiness/Low intensity	26.4 (4.4)	38.4 (4.4)	39.8 (4.4)	52.3 (4.4)	46.3 (4.4)	48.6 (4.4)	52.8 (4.4)	69.9 (4.4)	64.8 (4.4)	82.5 (5.8)
Happiness/High intensity	76.4 (5.5)	75.0 (5.5)	91.7 (5.5)	84.7 (5.5)	75.0 (5.5)	88.9 (5.5)	84.7 (5.5)	86.1 (5.5)	86.1 (5.5)	73.8 (7.2)
Sadness/Low intensity	34.5 (4.7)	41.1 (4.7)	55.3 (4.7)	58.9 (4.7)	60.7 (4.7)	58.3 (4.7)	63.7 (4.7)	62.5 (4.7)	61.9 (4.7)	75.5 (6.1)
Sadness/High intensity	72.9 (4.3)	68.7 (4.3)	89.6 (4.3)	94.8 (4.3)	78.1 (4.3)	85.4 (4.3)	86.4 (4.3)	88.5 (4.3)	94.8 (4.3)	85.7 (5.6)
Global emotion intensity score for low intensity	30.4 (17.7)	39.7 (19.7)	47.6 (22.0)	55.6 (21.8)	53.5 (18.6)	53.5 (22.4)	58.2 (20.3)	66.2 (16.5)	63.3 (15.2)	75.2 (17.2)
Global emotion intensity score for high intensity	74.6 (25.4)	71.9 (25.2)	90.6 (11.6)	89.7 (14.7)	76.6 (21.9)	87.1 (16.5)	85.6 (16.0)	87.3 (17.2)	90.4 (11.6)	79.0 (20.8)
Global emotion intensity score	52.5 (16.8)	55.8 (14.6)	69.1 (8.5)	72.7 (13.7)	65.0 (10.5)	70.3 (10.0)	71.9 (9.8)	76.8 (9.0)	76.9 (7.1)	77.1 (11.2)

## Data Availability

Data supporting the findings of this study are available from the corresponding author on a reasonable request.

## Supplementary Materials

The following are available online at https://www.mdpi.com/article/10.3390/children8121108/s1, Table S1: Results obtained for the Levene’s test for homogeneity of variances. Figure S1: Mean scores corrects (percentage) and standard deviations for the expression identification task (ID) according to the conditions Emotion (Happiness, Sadness, Anger, Surprise and Neutral) and Age Groups (4 to Adults). Figure S2: Mean scores corrects (percentage) and standard deviations for the expression matching task (Match) according to the conditions Emotion (Happiness, Sadness, Anger, Surprise and Neutral) and Age Groups (4 to Adults). Figure S3: Mean scores corrects (percentage) and standard deviations of global scores obtained in Emotion Expression tests according to the conditions Emotion (Happiness, Sadness, Anger, Surprise and Neutral) Task (Identification vs. Matching) and Age Groups (4 to Adults). Figure S4: Mean scores corrects (percentage) and standard deviations for the Facial Discrimination Task according to the conditions Emotion (Happiness, Sadness, and Neutral) and Age Groups (4 to Adults). The global expression discrimination score is also represented on this graph. Figure S5: Mean scores corrects (percentage) and standard deviations for each intensity in the Facial Discrimination Task according to the conditions Emotion (Happiness, Sadness, and Neutral), to the intensity (low vs. high) and Age Groups (4 to Adults). Figure S6: Mean scores corrects (percentage) and standard deviations of global scores obtained in the Facial Discrimination Task according to the intensity (low vs. high) and Age Groups (4 to Adults). Figure S7: The ability to attribute low intensity emotional expressions mediates, in a complementary manner, the relationship between age and the Global Identification score. The significant indirect effect (a x b = 0.14; 95% CI [0.07, 0.23] not containing zero which confirmed the existence of a mediation. Figure S8: The ability to attribute high intensity emotional expressions was not a mediator of the relationship between age and the Global Identification score. The non-significant indirect effect (a x b = −0.002; 95% CI [−0.04, 0.43] containing zero which confirmed the absence of an indirect mediation. Figure S9: The ability to attribute low intensity emotional expressions mediates, in a complementary manner, the relationship between age and the Global Matching score. The significant indirect effect (a x b = 0.24; 95% CI [0.14, 0.36] not containing zero which confirmed the existence of a mediation. Figure S10: The ability to attribute high intensity emotional expressions was not a mediator of the relationship between age and the Global Matching score. The non-significant indirect effect (a x b = −0.001; 95% CI [−0.03, 0.03] containing zero which confirmed the absence of an indirect mediation.
