# Supplementary material for: Sensitivity to Emotion Intensity and Recognition of Emotion Expression in Neurotypical Children
Source: Children (Basel). 2021 Dec 1;8(12):1108. doi: 10.3390/children8121108 (PMC8700579; doi:10.3390/children8121108)
Supplement: Supplementary file 1 [file children-08-01108-s001.zip › children-1442076-supplementary.pdf]

Supplementary material

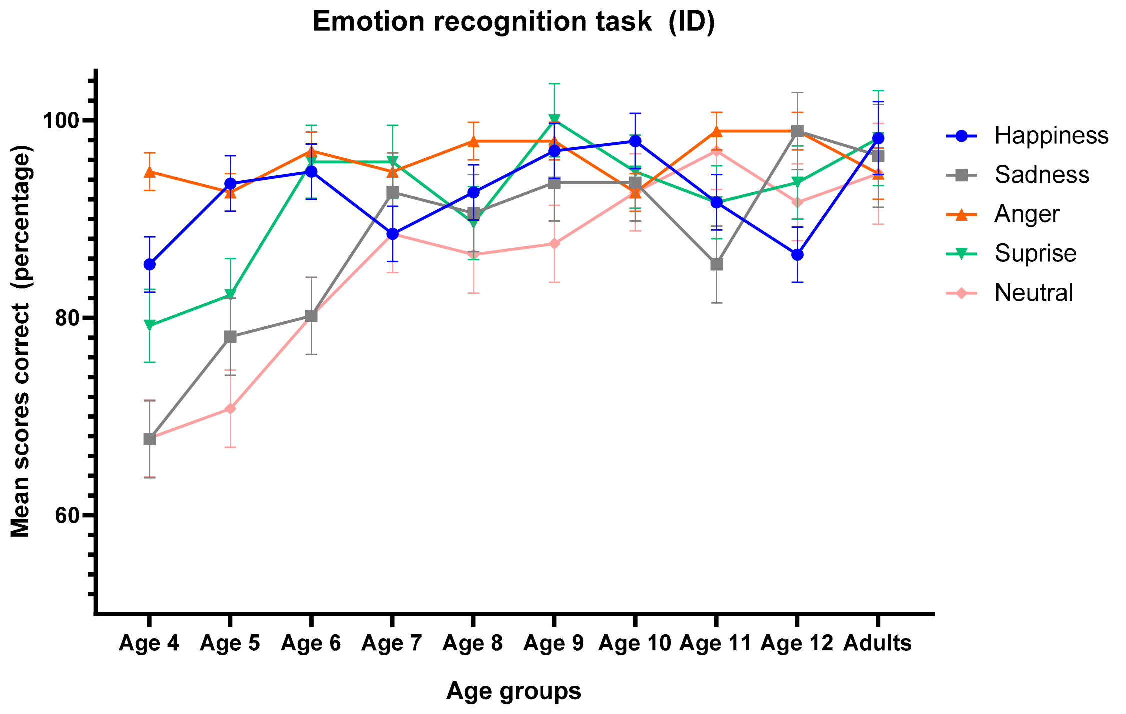

Figure S1. Mean scores corrects (percentage) and standard deviations for the expression identification task (ID) according to the conditions Emotion (Happiness, Sadness, Anger, Surprise and Neutral) and Age Groups (4 to Adults).

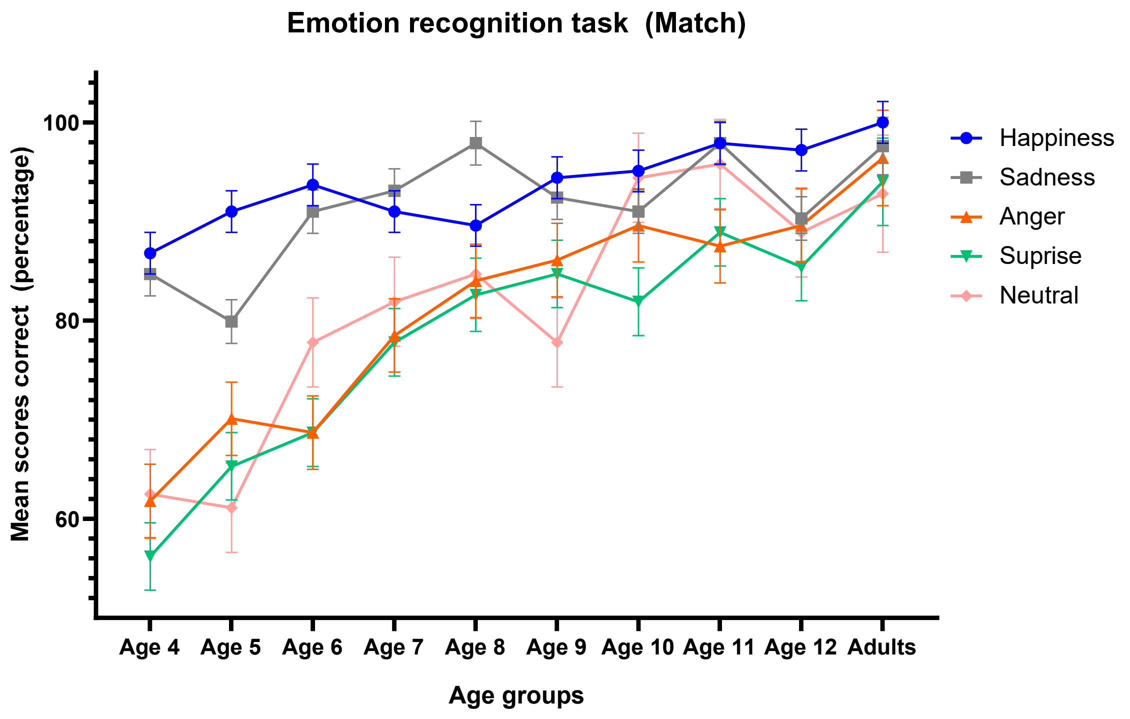

Figure S2. Mean scores corrects (percentage) and standard deviations for the expression matching task (Match) according to the conditions Emotion (Happiness, Sadness, Anger, Surprise and Neutral) and Age Groups (4 to Adults).

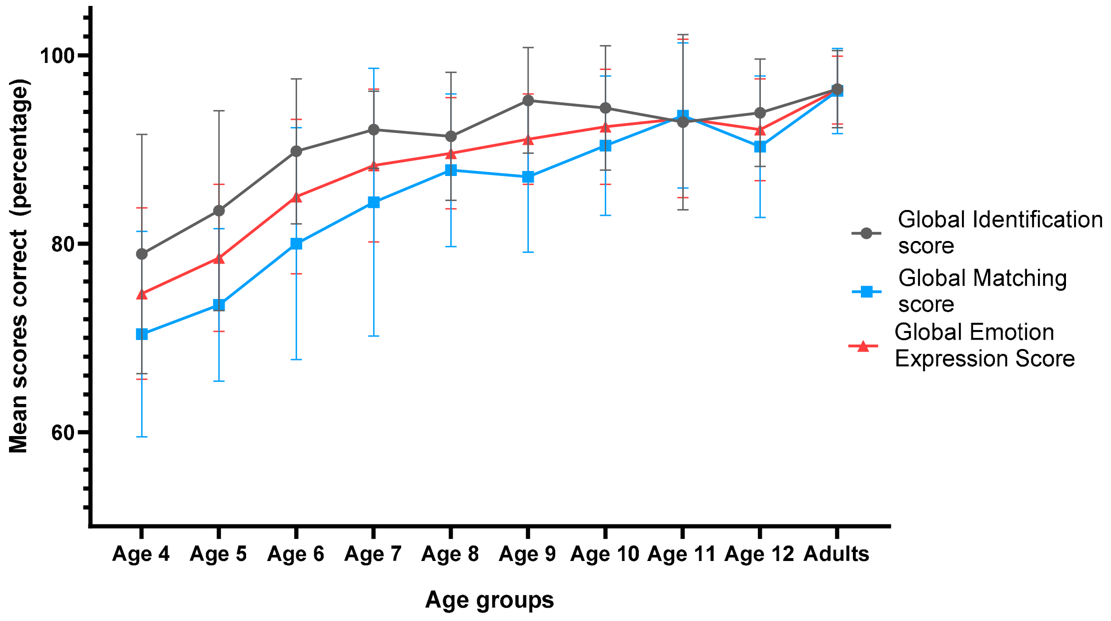

Figure S3. Mean scores corrects (percentage) and standard deviations of global scores obtained in Emotion Expression tests according to the conditions Emotion (Happiness, Sadness, Anger, Surprise and Neutral) Task (Identification vs Matching) and Age Groups (4 to Adults).

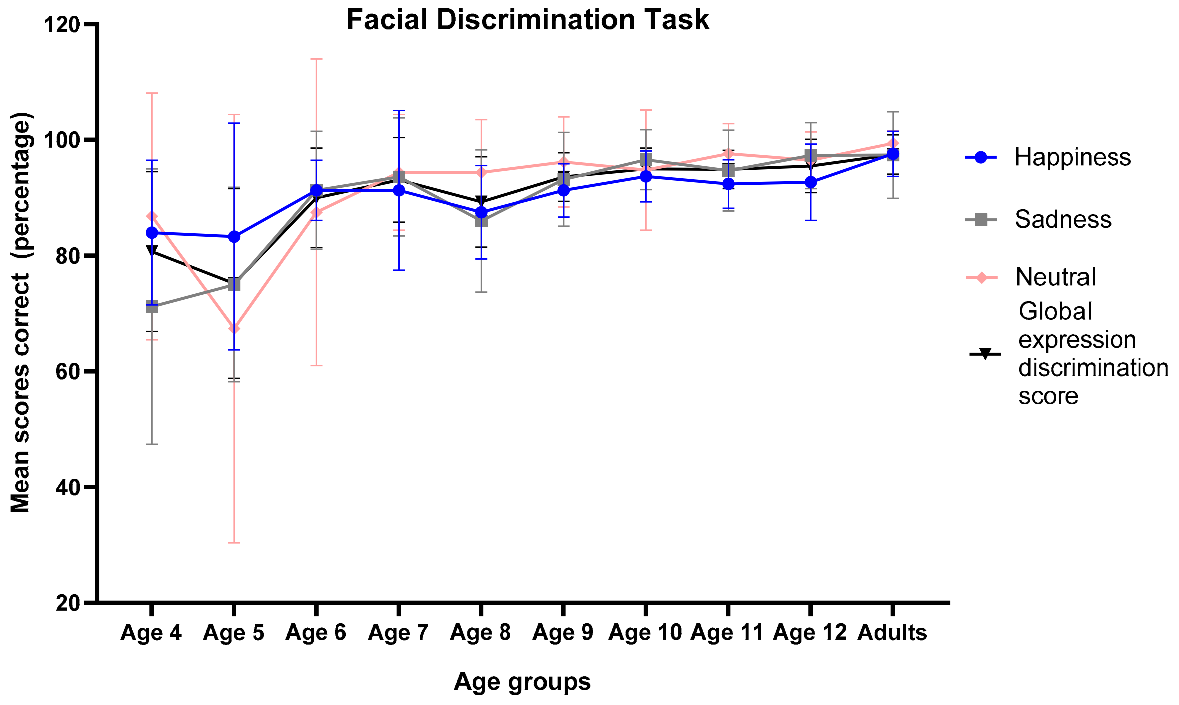

Figure S4. Mean scores corrects (percentage) and standard deviations for the Facial Discrimination Task according to the conditions Emotion (Happiness, Sadness, and Neutral) and Age Groups (4 to Adults). The global expression discrimination score is also represented on this graph.

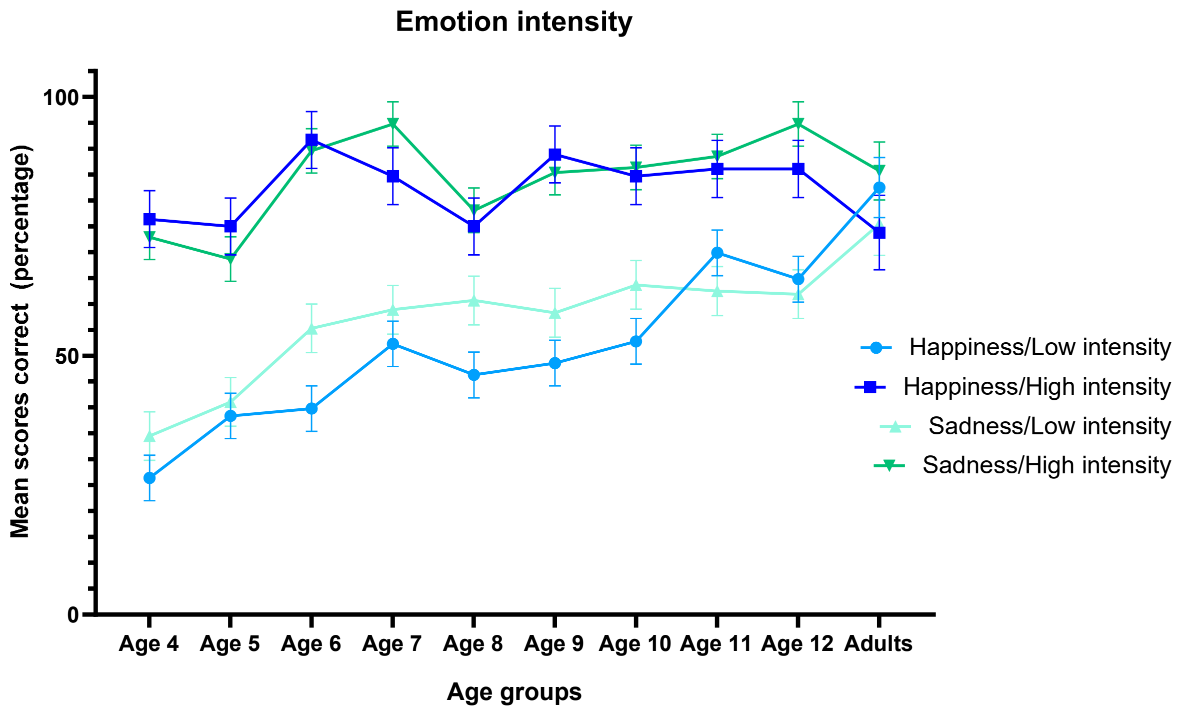

Figure S5. Mean scores corrects (percentage) and standard deviations for each intensity in the Facial Discrimination Task according to the conditions Emotion (Happiness, Sadness, and Neutral), to the intensity (low vs high) and Age Groups (4 to Adults).

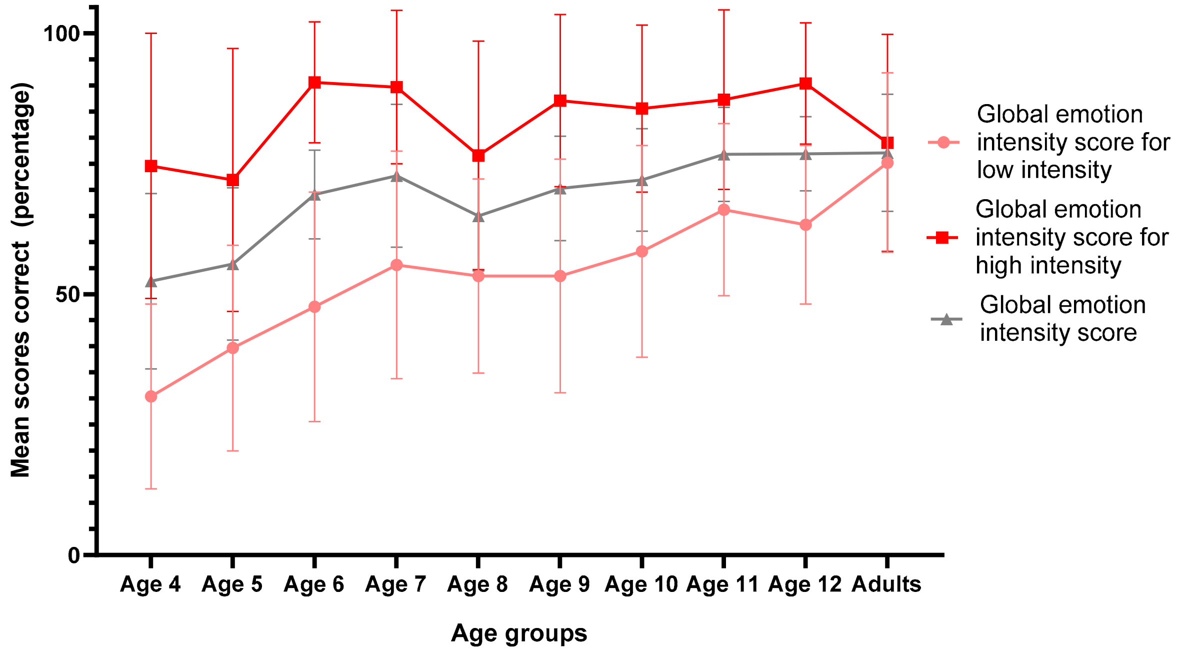

Figure S6. Mean scores corrects (percentage) and standard deviations of global scores obtained in the Facial Discrimination Task according to the intensity (low vs high) and Age Groups (4 to Adults).

Table S1. Results obtained for the Levene's test for homogeneity of variances.

Task	Variables	Ms Effect	F	p-value
Emotion Expression tests (ID task)	Happiness	510,804	7,62432	0,000000*
	Sadness	1274,587	10,77068	0,000000*
	Fear	260,258	6,49232	0,000000*
	Surprise	1294,048	9,14673	0,000000*
	Neutral	925,373	8,95135	0,000000*
	Total score	106,244	4,16731	0,000054*
Emotion Expression tests (Matching task)	Happiness	165,131	4,84299	0,000006*
	Sadness	197,225	4,91943	0,000005*
	Fear	230,906	1,81358	0,066740
	Surprise	326,422	4,09122	0,000068*
	Neutral	904,710	6,56939	0,000000*
	Total score	92,572	2,72299	0,004913*
Facial Discriminatio n Task	Happiness	321,809	6,86212	0,000000*
	Sadness	487,880	9,86974	0,000000*
	Neutral	2032,656	19,05683	0,000000*
	Global expression discrimination score	354,652	13,44377	0,000000*
	Happiness/low intensity	339,300	2,48418	0,010030*
	Happiness/high intensity	654,278	2,76847	0,004282*
	Sadness/low intensity	96,165	0,58159	0,811633
	Sadness/high intensity	545,501	3,64227	0,000284*
	Global emotion intensity score for low intensity	167,044	1,43262	0,174995
	Global emotion intensity score for high intensity	325,330	2,45396	0,010965*
	Global emotion intensity score	100,976	1,72915	0,083387

* indicated that the p-value < 0.05

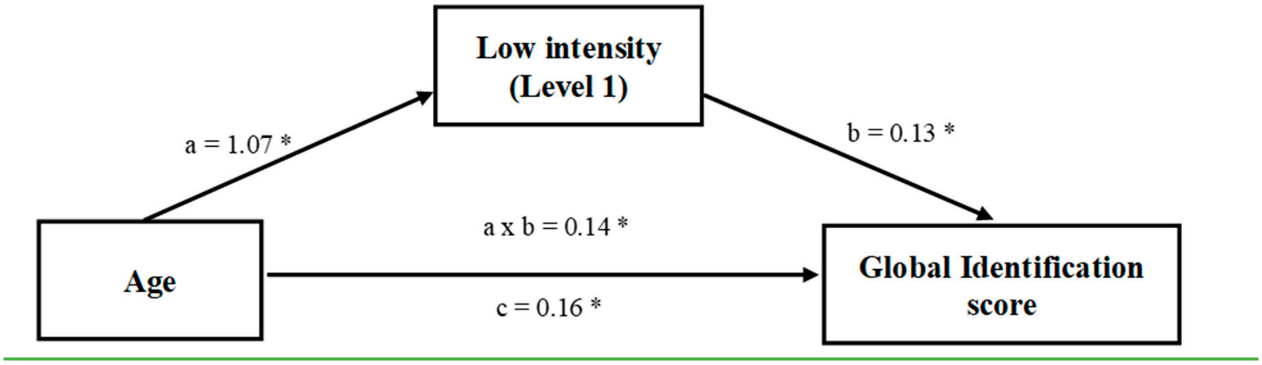

* Indicated that the parameter is significant ($p < 0.05$)

Figure S7. The ability to attribute low intensity emotional expressions mediates, in a complementary manner, the relationship between age and the Global Identification score. The significant indirect effect (a x b = 0.14; 95% CI [0.07, 0.23] not containing zero which confirmed the existence of a mediation.

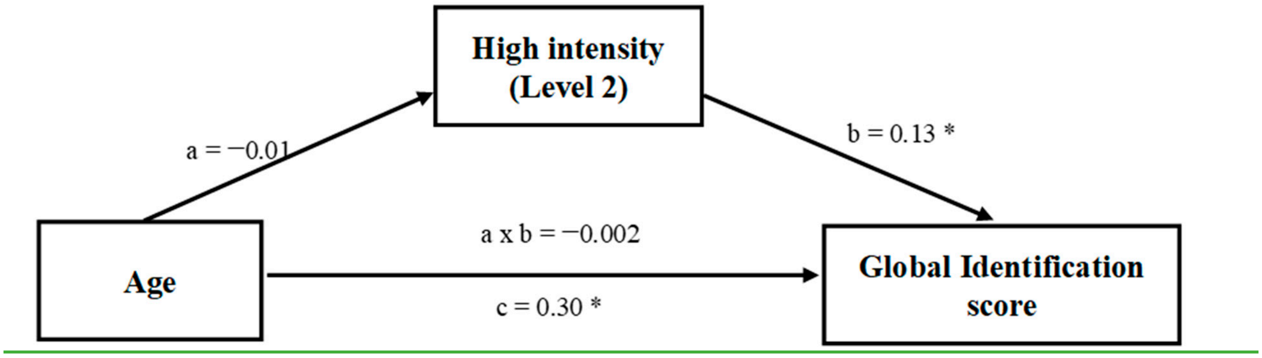

* Indicated that the parameter is significant ($p < 0.05$)

Figure S8. The ability to attribute high intensity emotional expressions was not a mediator of the relationship between age and the Global Identification score. The non-significant indirect effect (a x b = −0.002 ; 95% CI [−0.04, 0.43] containing zero which confirmed the absence of an indirect mediation.

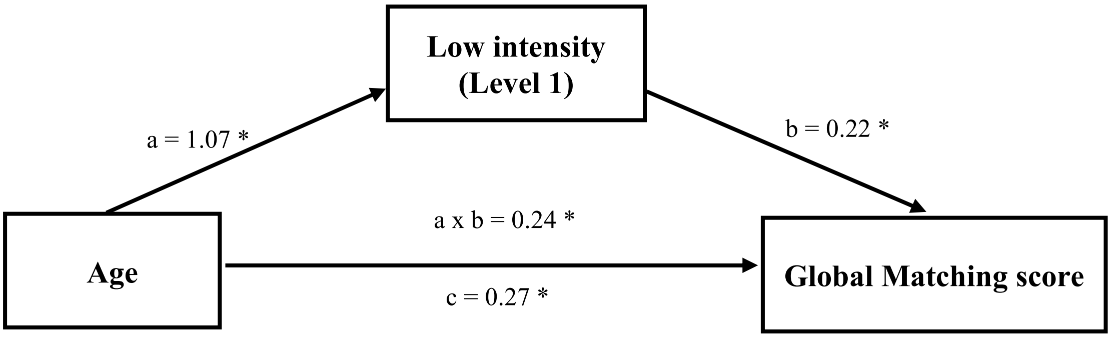

* Indicated that the parameter is significant ($p < 0.05$)

Figure S9. The ability to attribute low intensity emotional expressions mediates, in a complementary manner, the relationship between age and the Global Matching score. The significant indirect effect (a x b = 0.24; 95% CI [0.14, 0.36] not containing zero which confirmed the existence of a mediation.

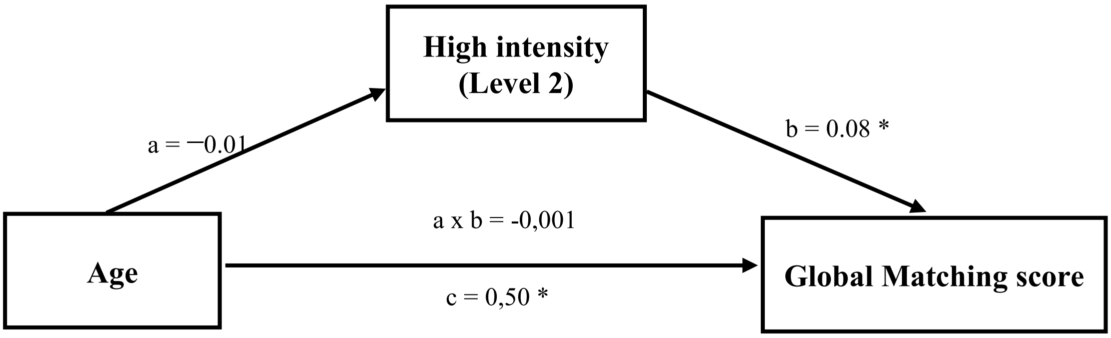

* Indicated that the parameter is significant ($p < 0.05$)

Figure S10. The ability to attribute high intensity emotional expressions was not a mediator of the relationship between age and the Global Matching score. The non-significant indirect effect (a x b = −0.001 ; 95% CI [−0.03, 0.03] containing zero which confirmed the absence of an indirect mediation.
